# Prevalence and risk factors of mortality among heart failure patients in low resource setting hospitals: a multicenter prospective observational study

**DOI:** 10.3389/fcvm.2024.1429513

**Published:** 2024-11-21

**Authors:** Firomsa Bekele, Tadesse Sheleme, Tesfaye Tsegaye, S. Angala Parameswari, Manjoor Ahamad Syed, Lalise Tafese, Wubishet Gezimu

**Affiliations:** ^1^ Department of Pharmacy, Institute of Health Science, Wallaga University, Nekemte, Ethiopia; ^2^ Department of Pharmacy, College of Health Science, Mattu University, Mattu, Ethiopia; ^3^ Department of Health Informatics, College of Health Science, Mattu University, Mattu, Ethiopia; ^4^ Department of Nursing, College of Health Science, Mattu University, Mattu, Ethiopia

**Keywords:** heart failure, mortality, associated factors, Ethiopia, prevalence

## Abstract

**Background:**

Heart failure is a significant worldwide health problem that leads to mortality. Therefore, this study aimed to evaluate treatment outcomes and associated factors of heart failure patients who were admitted to hospitals in the southwest of Ethiopia.

**Methods and participants:**

A multicenter prospective observational study was conducted from 1 February to 1 August 2021. Drug therapy problems were assessed as per the Cipolle, Strands, and Morley drug therapy problems classification method. The drug therapy was registered by using the drug-related problem registration format. The results of logistic regression analysis was interpreted as crude odds ratio and adjusted odds ratio (AOR) at 95% confidence interval (CI) to determine the association between dependent and independent variables.

**Results:**

In our study settings, a total of 205 (85.1%) heart failure patients showed improvement and 36 (14.9%) died at hospital discharge. Being ≥65 years (AOR = 7.14, 95% CI: 2.04–.25.01, *P* = 0.002), a previous hospitalization (AOR = 6.20, 95% CI: 1.81–21.21, *P* = 0.004), and the presence of medication-related problems (AOR = 3.65, 95% CI: 1.13–11.73, *P* = 0.03) were the predictors of mortality.

**Conclusion:**

The prevalence of in-hospital mortality among heart failure patients was found to be high. Previous hospitalization, older age, and the presence of drug therapy problems were the predictors of mortality among heart failure patients. Therefore, proper attention should be given to the management of elderly and re-admitted heart failure patients in addition to their regular care. In addition, hospitals should implement clinical pharmacy services to address any drug-related problems.

## Background

Heart failure (HF) is a multifactorial clinical condition defined by the heart's diminished capacity to pump and/or fill with blood (1). The clinical presentations of the HF include dyspnea, exhaustion, low exercise tolerance, and fluid retention (2, 3).

There are 64.34 million cases of heart failure worldwide (4). Heart failure is a well-known public health issue that places a heavy burden on patients and healthcare systems (5), with a prevalence of 6.5 million in the United States (US) (6).

Africa is witnessing an increase in the prevalence of HF, especially in sub-Saharan Africa (SSA) (7). There are various reports of the prevalence of heart failure across sub-Saharan Africa. It was 30% in Cameroon, it ranges from 9.4% to 42.5% in Nigeria, and it ranges from 25.6% to 28.6% in Togo (8). According to a study done in Ethiopia, 23.9% of people have HF, making it one of the most prevalent cardiovascular disorders (9), whereas in Burkina Faso it accounts for 17.62% (10).

Data from the eight SSA countries have shown that up to 75% of heart failure cases were non-ischemic in origin (7). Several studies have shown that the most common causes of heart failure in SSA are cardiomyopathies and hypertension (11). Patients with heart failure in sub-Saharan Africa were the youngest at onset, and most likely to be in New York Health Association functional class IV compared with those from Asia, the Middle East, and South America (7).

Even in affluent nations, HF is a primary cause of hospitalization, and treatment outcomes are still subpar (5, 12). Consequently, the goals of HF therapy are to increase functional ability, decrease hospitalization, and prolong life (13).

Hospitalizations and readmissions for heart failure are frequent, and residual mortality is still high even with the intensive use of angiotensin converting enzyme inhibitors (ACEIs), angiotensin receptor blockers, beta-blockers, and aldosterone antagonists (14, 15). People with HF report a generally lower quality of life and considerable negative consequences on their ability to manage themselves at home (16).

Patients need medication treatment for both HF and related disorders since HF is the end-stage of several cardiovascular diseases and their inherent risk factors (17). Polypharmacy in the management of heart failure patients has been linked to worse treatment results, medication interactions, improper drug prescriptions, poor adherence, and an increased risk of drug-related problems (DRP) (18, 19). In lower middle income countries, HF patients' poor medication adherence significantly increases the chance of mortality and hospital readmissions (20). Heart failure–associated mortality increases among patients having medication-related problems (21).

For many years, heart failure syndrome has been acknowledged as a major factor in the burden of cardiovascular illness in sub-Saharan Africa (22). Hospital mortality in Angola was 23% (23). In Nigeria, the overall death rate was 4.3% (24). In Mozambique, the composite death rate was 21.1% (25). The death rate in Kenya was 7.6% (26). The total incidence of all-cause mortality in Uganda was 3.58 per 1,000 people (27).

Heart failure patients having lower left ventricular ejection fraction (EF) have poor treatment outcomes. In spite of this, there is a dearth of prospective and multicenter evidence regarding the outcomes and predictors of heart failure treatment. Thus, the study's objective was to evaluate the treatment outcome and associated factors of heart failure patients in southwest Ethiopia hospital.

## Methodology

### Study area, design, and period

A multicenter prospective observational study was conducted at five hospitals in the Iluababor and Buno Bedele zone, southwestern Ethiopia, namely, Mettu Karl Comprehensive Specialized Hospital (MKSCH), Bedele Hospital (BH), Darimu Hospital (DH), Chora Hospital (CH), and Didesa Hospital (DIH) from 1 February to 1 August 2021.

### Study participants and eligibility criteria

Patients who were over the age of 18, diagnosed with HF, hospitalized for more than 48 h, and receiving HF treatment during the study period were included in the study. Patients who declined to participate, who were transferred to another hospital, whose doctors did not confirm their diagnosis of congestive heart failure (CHF), and who chose to discharge themselves were excluded. CHF was diagnosed based on different investigations like chest X-ray, electrocardiogram, ejection fraction, and echocardiogram.

### Study variables and outcome endpoints

The primary treatment outcome included mortality, improvement in signs and symptoms, ejection fraction, HF stage, and New York Heart Association (NYHA) functional class. The patients HF stage and class were based on NYHA functional classification and American College of Cardiology (ACC)/American Heart Association (AHA) stages (28).

On a daily basis, patients were observed to evaluate if the symptoms and indicators of HF had disappeared. Physicians conducted a thorough echocardiographic evaluation of regional wall anomalies, ventricular function, and valvular structure and function.

Drug therapy problems (DTPs) were assessed as per the Cipolle, Strands, and Morley drug therapy problems classification method (29). Mortality was evaluated at discharge from hospital from 1 February to 1 August 2021.

### Sample size and sampling technique

Single population proportion formula was used to calculate the required sample size by considering the following assumptions: *n* is the required sample size, *p* is the hospital mortality and found to be 17.2% (0.172) (21), *Z* is the standardized normal distribution value at 95% confidence interval (CI) = 1.96, and *d* is the margin of error of 5% (0.05).

$$\frac{n={\left(Z\alpha /2\right)}^{2}p\;(1-p)}{d^{2}}$$

$$\frac{n={\left(1.96\right)}^{2}(0.172)(0.828)=218.8-219}{{\left(0.05\right)}^{2}}$$

A 10% loss to follow-up yielded a final sample size of 241. The study subjects were selected by proportional allocation based on the number of HF patients that the respective hospitals contained in their medical ward: 82 patients from MKSCH, 51 from BH, 43 from DH, 30 from CH, and 35 from DIH were included. The subjects were chosen by using the consecutive sampling technique from 1 February to 1 August 2021.

### Data collection process and management

A questionnaire that was created after studying several works of literature was used to collect the data. Through patient interviews, information was gathered about the patients' socio-demographic traits, medication compliance, smoking status, alcohol intake, and chewing gum behavior. For the purpose of gathering data, five nurses and five pharmacists were enlisted. The task of overseeing the data collection procedure was given to five physicians and five pharmacists. The lead investigator and supervisor constantly monitored the procedure of gathering data on the spot. Following data collection, the lead investigator assessed the suitability of medical therapy using a variety of sources, including guidelines and references from Medscape, Up-to-date, Lexicom, and Micromedex. DTP was registered using the DRP registration format.

### Data processing and analysis

The data were analyzed using Statistical Package for Social Sciences (SPSS) version 23. By utilizing odds ratio (OR) (95% CI), multivariable and binary logistic regression were utilized to identify the factors. The categorical variables were compared using the chi-square test. If the *P*-value is <0.05, we finally announce the relationships between the treatment outcome and the independent variables.

### Operational definitions

Treatment outcome: The accomplishment of a predetermined goal, such as improvement or death (21).

Poor outcome: HF-related hospital mortality (21).

Good outcome: When patients are discharged having improved (21).

Drug therapy problem/drug-related problems: Drug interactions, unsuitable indications and dosages, non-adherence, adverse drug reactions (ADRs), and poor pharmacological therapy are all included in this study (30).

Preserved EF: A clinical diagnosis of HF and an LVEF of >40% (31).

Reduced EF: A clinical diagnosis of HF and an LVEF of ≤40% (31).

## Results

### Socio-demographic and life style characteristics of the study participants

From the 241 HF patients admitted to the medical wards of southwestern Ethiopian hospitals, 138 (57.3%) were males. Half of the patients [125 (51.9%)] were in the age range of 45–64 years, while the mean and standard deviation (SD) of the patients age was 52.09 ± 16.75. Regarding their area of residence, 131 (54.4%) were from rural areas. A total of 172 (71.4%) were married and 120 (49.8%) of them were farmers. Regarding their lifestyle, 53 (21.99%) of them drunk alcohol, 23 (9.54%) used tobacco, 82 (34.02%) chewed khat, 139 (57.68%) were physically active, and 145 (60.17%) consumed a salt (Table 1).

**Table 1:** Socio-demographic and life style factors of CHF patients admitted to the medical wards of southwestern Ethiopian hospitals from 1 February to 1 August 2021.

Variables	Total (*n* = 241)	Frequency (*n*)	Percentage
Age (years)	18–44	63	26.1
	45–64	125	51.9
	≥65	53	22
Sex	Male	138	57.3
	Female	103	42.7
Residence	Urban	110	45.6
	Rural	131	54.4
Marital status	Married	172	71.4
	Single	32	13.3
	Divorced	15	6.2
	Widowed	22	9.1
Occupation	Farmer	120	49.8
	Merchant	46	19.1
	Govt employee	14	5.8
	Housewife	39	16.2
	Student	14	5.8
	Daily labor	8	3.3
Level of education	No formal education	118	49.0
	Primary	75	31.1
	Secondary	37	15.4
	College and above	11	4.6
Drunk alcohol	Yes	53	21.99
	No	188	78.0
Tobacco use	Yes	23	9.54
	No	218	90.46
Chew khat	Yes	82	34.02
	No	159	65.98
Physically active	Yes	139	57.68
	No	102	42.32
Consumed a salt	Yes	145	60.17
	No	96	39.83

### The clinical characteristics of the study respondents

Over the study period, about two-third [162 (67.2%)] of the HF patients were categorized under stage C and 147 (61.0%) of them were under NYHA class IV. A total of 131 (54.4%) patients had a previous history of admission for HF. About half 126 (52.3%) of the HF patients presented with two or more than two comorbid diseases, and most of them had hypertension [48 (19.92%)] (Table 2). The average length of hospital stay (LOS) was 11.13 ± 8.07 days. In relation to chronic kidney disease (CKD), the mean ± SD of estimated glomerular filtration rate (eGFR) was 51 + 12.53 ml/min/1.73 m^2^.

**Table 2:** Clinical characteristics of CHF patients admitted to the medical wards of southwestern Ethiopian hospitals from 1 February to 1 August 2021.

Variables	Total (*n* = 241)	CHF category	
		HFrEF	HFpEF
CHF class	I	8 (3.3)	3 (37.5) 5 (62.5)
	II	33 (13.7)	16 (48.48) 17 (51.52)
	III	53 (22)	20 (37.74) 33 (62.26)
	IV	147 (61.0)	57 (38.78) 90 (61.22)
CHF stage	A	21 (8.7)	8 (38.09) 13 (61.91)
	B	41 (17.0)	30 (73.17) 11 (26.83)
	C	162 (67.2)	102 (62.96) 60 (37.04)
	D	17 (7.1)	13 (76.47) 4 (23.53)
Admission history	Yes	131 (54.4)	81 (61.83) 50 (38.17)
	No	110 (45.6)	54 (49.09) 56 (50.91)
Presence of comorbidity	Yes	126 (52.3)	100 (79.37) 26 (20.63)
	No	115 (47.7)	50 (43.48) 65 (56.52)
Types of comorbidity	Hypertension	48 (19.92)	30 (62.5) 18 (37.5)
	Diabetes mellitus	31 (12.9)	14 (45.16) 17 (54.86)
	CKD	28 (11.62)	18 (64.29) 10 (35.71)
	Stroke	10 (4.1)	6 (60.0) 4 (40.0)
	Others^a^	17 (7.1)	7 (41.18) 10 (58.82)

^a^ Others include asthma, hyperlipidemia, pneumonia, arrhythmia, malaria, dyspepsia, and deep vein thrombosis.

### The most commonly prescribed drugs

The most commonly prescribed medications were furosemide [210 (87.14%)], spironolactone [96 (39.83%)], and enalapril [83 (34.44%)], whereas statins [4 (1.66%)] were the least likely prescribed medications for CHF patients (Table 3).

**Table 3:** The commonly prescribed heart failure medication at the medical wards of southwestern Ethiopian hospitals from 1 February to 1 August 2021.

Drugs	Frequency (*n*)	Percentage
Furosemide	210	87.14
Spironolactone	96	39.83
Enalapril	83	34.44
Metoprolol	39	16.18
Digoxin	37	15.35
Acetylsalicylic acid	31	12.86
Atenolol	27	11.20
Hydrochlorothiazide	21	8.71
Statins	4	1.66

### The etiology of heart failure

In our study, the most common causes of heart failure was dilated cardiomyopathy [56 (23.24%)], coronary artery disease [48 (19.92%)], and hypertensive heart disease [35 (14.52%)], whereas congenital heart disease [12 (4.98%)] was the least likely cause (Table 4).

**Table 4:** The common etiologies of heart failure patients admitted to the medical wards of southwestern Ethiopian hospitals from 1 February to 1 August 2021.

Causes of heart failure	Frequency (*n*)	Percentage
Dilated cardiomyopathy	56	23.24
Coronary artery disease	48	19.92
Hypertensive heart disease	35	14.52
Rheumatic heart disease	32	13.28
Cor pulmonale	25	10.37
Ischemic cardiomyopathy	17	7.05
Non-rheumatic valvular heart disease	16	6.54
Congenital heart disease	12	4.98

### Prevalence and types of drug therapy problems

Over the study period, total of 165 (68.5%) drug therapy problems were identified, whereas 76 (31.5%) patients were appropriately treated. The most commonly occurred drug therapy problems were the need for additional drug therapy [41 (17.02%)], too low dose [31 (12.86%)], and ineffective drug therapy [28 (11.62%)], whereas adverse drug reaction [8 (3.32%)] was the least likely occurred drug therapy problems (Table 5).

**Table 5:** Types of drug therapy problems of the heart failure patients admitted to the medical wards of southwestern Ethiopian hospitals from 1 February to 1 August 1.

Types of drug therapy problems	Frequency (*n*)	Percentage
Ineffective drug therapy	28	11.62
Dose too low	31	12.86
Dose too high	23	9.54
Unnecessary drug therapy	18	7.46
Adverse drug reaction	8	3.32
Needs additional drug therapy	41	17.02
Non-compliance	16	6.64

### Magnitudes and determinants of mortality among HF patients

Over the study period, a total of 205 (85.1%) HF patients showed improved and 36 (14.9%) died. The mortality rates in MKSCH, BH, DH, DIH, and CH were 12 (33.33%), 9 (25%), 6 (16.67), 5 (13.89%), and 4 (11.11%), respectively. Patients aged ≥65 years were seven times more likely to die than younger patients [adjusted odds ratio (AOR) = 7.14, 95% CI: 2.04–25.01, *P* = 0.002]. Heart failure patients who had a previous admission were six times more likely to die than naïve patients (AOR = 6.20, 95% CI: 1.81–21.21, *P* = 0.004). Finally, patients who had medication-related problems were 3.65 times more likely to die than patients who did not have any drug therapy problems (AOR = 3.65, 95% CI: 1.13–11.73, *P* = 0.03) (Table 6).

**Table 6:** Bivariable and multivariable logistic regression analysis of factors associated with the treatment outcomes of CHF patients admitted to the medical wards of southwestern Ethiopian hospitals from 1 February to 1 August 2021.

Variables	Category	Treatment outcome		COR (95% CI)	AOR (95% CI)	*P*-value
		Improved	Died			
Age (years)	18–44	59 (28.78%)	4 (11.11%)	1	1	0.20
	45–64	118 (57.56%)	7 (19.44%)	0.875 (0.25–3.11)	0.44 (0.11–1.78)	0.25
	≥65	28 (13.66%)	25 (69.44%	13.17 (4.18–41.48)	7.14 (2.04–25.01)	0.002
Drug therapy problem	Yes	134 (65.37%)	31 (86.11%)	3.29 (1.22–8.82)	3.65 (1.13–11.73)	0.03
	No	71 (34.63%)	5 (13.89%)	1	1	
Residency	Urban	98 (47.80%)	12 (33.33%)	1	1	0.202
	Rural	107 (52.20%)	24 (66.67%)	1.83 (0.87–3.86)	1.85 (0.72–4.74)	
Drinking alcohol	Yes	42 (20.49%)	11 (30.56%)	1.71 (0.78–3.75)	1.47 (0.52–4.13)	0.47
	No	163 (79.51%)	25 (69.44%)	1	1	
Salt intake	Yes	120 (58.54%)	25 (69.44%)	0.62 (0.29–1.33)	2.11 (0.76–5.89)	0.15
	No	85 (41.46%)	11 (30.66%)	1	1	
Admission history	Yes	101 (49.27%)	30 (83.33%)	5.15 (2.06–12.89)	6.20 (1.81–21.21)	0.004
	No	104 (50.73%)	6 (16.67%)	1	1	
Comorbidity	Yes	101 (49.27%)	25 (69.44%)	2.34 (1.09–5.01)	0.65 (0.21–1.99)	0.45
	No	104 (50.73%)	11 (30.56%)	1	1	

AOR, adjusted odds ratio; CI, confidence interval; COR, crude odds ratio.

## Discussion

In our study, the magnitude of mortality in congestive heart failure was 36 (14.9%). This was higher than the study conducted in the US (3.8%) (32) and Brazil (11.2%) (33), and lower than Spain (33.2%) (34), Saudi Arabia (19.09%) (35), and Tikur Anbessa Specialized Hospital (TASH) (17.2%) (21). As compared with other sub-Saharan African nations, the mortality rate was higher than Kenya (7.6%) (26), Nigeria (4.3%) (24), and Uganda (3.58 per 1,000 person-days) (27), and lower than the studies done in Tanzania (25.4%) (36), Burkina Faso (31%) (10), and Angola (23%) (23). The variety of outcomes reported might be due to the difference in the quality of HF management, methodology, and multicenter coverage of the study participants.

Regarding the prediction of mortality among HF patients, patients aged ≥65 years were seven times more likely to die than younger patients (AOR = 7.14, 95% CI: 2.04–.25.01, *P* = 0.002). This was consistent with the study conducted at the US (32), Canada (37), Spain (34), Brazil (33), Korea (38), Uganda (27), and Saudi Arabia (26). However, the study conducted in Angola showed that mortality was high among young and middle-aged groups (23). This is due to the fact that as the age increased, the organ functions reduced, impairing the pharmacokinetics of the drugs, and elder patients presented with a variety of comorbidities that might impose the burden of worsening outcomes in HF patients.

The HF patients who had a previous admission were six times more prone to die than naïve patients (AOR = 6.20, 95% CI: 1.81–21.21, *P* = 0.004). This was in line with the finding from the International Congestive Heart Failure (INTER-CHF) study (39) and studies from Brazil (33) and Korea (38). Similarly, in North Middlesex University Hospital and the Acute Heart Failure Global Registry of Standard Treatment (ALARM-HF) global survey, long-term mortality was significantly lower in single admitters vs. re-admitters (*p* < 0.0001) (40, 41). The study conducted in Burkina Faso also revealed that the mortality was lower among newly admitted heart failure patients (10).

In our study the management of the decompensated heart failure was more complex and lifesaving positive inotropic medications were unavailable that results in the risk of mortality than HF naïve patients.

Over the study period, total of 165 (68.5%) drug therapy problems were identified, whereas 76 (31.5%) patients were appropriately treated. This was consistent with the study conducted at Jimma Medical Center and medical wards of Mettu Karl Hospital, Bedele General Hospital (BGH), and Darimu General Hospital (42, 43). The most commonly occurred drug therapy problems were the need for additional drug therapy [41 (17.02%)], too low dose [31 (12.86%)], and ineffective drug therapy [28 (11.62%)]. Patients who had medication-related problems were 3.65 times more prone to die than patients who did not have any drug therapy problems (AOR = 3.65, 95% CI: 1.13–11.73, *P* = 0.03). This is similar with the finding from TASH (21) and a systematic review and meta-analysis study done by Bekele et al. (44). Drug-related problem registration format was used to identify and record different types of drug-related problems.

There was no association between the stages and class of HF in our study area with the treatment outcomes of HF patients. Despite this, the advanced stages and HF classes were predictors of mortality according to the INTER-CHF study (39) and studies conducted in South India (45) and Saudi Arabia (35). In Uganda, NYHA class IV was the predictor of mortality (27). Therefore, the patients on advanced stage HF should not be ignored.

To improve heart failure outcomes in resource-limited settings, management of heart failure should be individualized based on each patient's clinical characteristics, preferences, and comorbidities. In addition, to improve the medication compliance, educating and counseling patients to increase their knowledge about HF therapy is paramount (46). Finally, self-care is viewed as the cornerstone of HF therapy and entails essential practices that have been shown to improve HF clinical outcomes. These include adjusting one's lifestyle by taking prescribed medications as directed, sodium diet restriction, exercising, reducing liquid intake, and routinely weighing oneself (47).

The three most common causes of heart failure in our study were hypertensive heart disease, coronary artery disease, and dilated cardiomyopathy. In Tanzania, the most often found cause of heart failure was uncontrolled hypertension (11). In Burkina Faso, the two most common causes of HF were idiopathic dilated cardiomyopathy (19.80%) and hypertensive heart disease (50.34%) (10).

In contrast, sepsis, congenital heart disease, severe anemia, and respiratory tract infections were the leading causes of heart failure in Nigeria (48). In Kenya, the common causes were infection, anemia, rheumatic heart disease, and congenital heart disease (27). The variety of heart failure causes might be due to the different study participants and the race of the included patients.

As a strength, the study was a prospective and multicenter study that extensively followed the patients. In addition, the frequency of medication-related issues and their effect on congestive heart failure treatment results were evaluated.

### The limitation of the study

The study only assessed the hospital mortality and some investigations like computed tomography scan was not available. The absence of this equipment might hinder the diagnosis and identification of the different causes of heart failure. The government should donate this machine to assist in rapid diagnosis of heart failure.

In addition, the data were collected during the COVID-19 pandemic that could impose a fear on data collectors. As the result, data collectors might not have addressed heart failure patients who had COVID-19. Therefore, personnel protective equipment should be available for all data collectors.

## Conclusion

The prevalence of mortality in HF patients was found to be high and more than two-third of the patients presented with one or more drug-related problems. The most commonly prescribed medications were furosemide, spironolactone, and enalapril. Over the study period, about two-third of the HF patients were categorized under HF stage C and NYHA class IV.

Regarding the treatment outcomes of HF, previous hospitalization, older age, and the presence of drug therapy problems were the predictors of mortality among HF patients. Inappropriate use of anti-heart failure medication was reported. Therefore, the patient should comply with the recommended guideline. In addition to usual care of the HF patients, due attention should be given in the management of elderly and re-admitted HF patients. Conversely, the clinical pharmacy services should be established in hospitals to tackle any drug-related problems in our study area. Clinical pharmacists should be assigned into medical wards and involved in daily patient rounds with physicians. The pharmaceutical care assessment checklist should be filled and evaluated by senior clinical pharmacy specialists for the presence of any medication-related problems.

## Data Availability

The original contributions presented in the study are included in the article/Supplementary Material, further inquiries can be directed to the corresponding author.
